# Video-Assisted Thoracoscopic Lung Biopsy in Critically Ill Patients With Hematologic Malignancy and Acute Respiratory Distress Syndrome: A Case Series Report

**DOI:** 10.1177/2324709620912101

**Published:** 2020-03-19

**Authors:** Elizabeth Arrieta, Saveria Sangiovanni, Juan Esteban Garcia-Robledo, Mauricio Velásquez, Luz Fernanda Sua, Liliana Fernández-Trujillo

**Affiliations:** 1 Fundación Valle del Lili, Cali, Colombia; 2 Universidad Icesi, Cali, Colombia

**Keywords:** hematologic malignancies, acute respiratory distress syndrome, video-assisted thoracoscopic, lung biopsy, case series

## Abstract

Pulmonary complications are prevalent among patients with hematologic malignancies, who are at high risk of developing acute respiratory distress syndrome (ARDS). Although diffuse alveolar damage is considered the diagnostic hallmark of ARDS, there are plenty of other non–diffuse alveolar damage etiologies that can mimic ARDS and benefit from a specific therapy, therefore correcting the underlying cause. When the etiology remains unclarified despite noninvasive procedures, a surgical lung biopsy (either open via thoracotomy or video-assisted thoracoscopic surgery [VATS]) may be warranted. However, the role of surgical lung biopsy has not been extensively studied in patients with hematologic malignancy and ARDS and so doubt exists about the risk-benefit relationship of such procedures. In this article, we report a series of 8 critically ill patients with hematologic malignancies and ARDS, who underwent VATS lung biopsy, in a specialized institution in Cali, Colombia, from 2015 to 2019, with special emphasis on its diagnostic yield, modifications in treatment protocol, and safety. VATS lung biopsy is a minimally invasive procedure that appears to be a relatively safe with few postoperative complications and minimal perioperative mortality. It has a high diagnostic yield, resulting in a modification of treatment in a nondepreciable percentage of patients. However, this subset of patients was critically ill, with a high risk of mortality, and the lung biopsy did not appear to affect in this aspect. Future randomized controlled trials are needed to further clarify this topic.

## Introduction

Pulmonary complications are prevalent among patients with hematologic malignancies,^1^ who are at high risk of developing acute respiratory distress syndrome (ARDS). The latter is a prevalent and lethal/disabling syndrome, whose etiological diagnosis and treatment is a challenge. ARDS is diagnosed using clinical criteria, specifically the Berlin definition proposed in 2012. However, from a histopathological point of view, diffuse alveolar damage (DAD) is considered the diagnostic hallmark. Nevertheless, there are many other non-DAD etiologies that can present as ARDS and benefit from a specific therapy, which is of relevance since the cornerstone of ARDS treatment is the identification and correction of the underlying cause.^2^

Reaching a specific diagnosis in this population is challenging due to patient’s instability, variable diagnostic yield of available tools, aberrant response to inflammatory processes, risk-benefit relationship of invasive procedures,^1^ and so on, which explains why it is typically delayed or not done at all. When the etiology remains unclarified, the most common procedure is to initiate empiric therapy, and although patients tend to respond, there is a small fraction that do not.^2,3^ This set of patients may benefit from a surgical lung biopsy, either open via thoracotomy or through video-assisted thoracoscopic surgery (VATS), to guide therapy when noninvasive diagnostic procedures have been performed and uncertainty persists. Although a recent meta-analysis performed with data of critically ill, mechanically ventilated patients showed that open lung biopsy yielded a wide range of diagnosis and that treatment was changed in 78% of subjects,^4^ other studies have not been able to show a substantial benefit in this population. Furthermore, the role of surgical lung biopsy has not been extensively studied in patients with hematologic malignancy and ARDS and so doubt exists about the risk-benefit relationship of such procedures.^3^

In this article, we aim to describe a series of 8 critically ill patients with hematologic malignancies and ARDS, who underwent VATS lung biopsy, with special emphasis on its diagnostic yield, modifications in treatment protocol, and safety.

## Methods

We performed a retrospective analysis of 8 patients with hematologic malignancies and ARDS, who had been admitted to the intensive care unit (ICU) in a specialized institution in Cali, Colombia, from 2015 to 2019, on whom VATS lung biopsy was performed. We included patients with a diagnosis of ARDS using the Berlin criteria, with a thoracic computed tomography (CT) scan showing pulmonary infiltrates, who had negative microbiological and immunological studies, normal bronchoalveolar lavage (BAL), and unremarkable findings in fibrobronchoscopy prior to the performance of the lung biopsy. Patients who died before biopsy results were excluded. All biopsies were performed in the operating room under general anesthesia. Selective intubation was performed with a double-lumen tube. Then a single-port VATS approach was chosen with the use of staplers. For every patient, a multidisciplinary board took place to decide the best site from which to take the lung sample, taking into consideration each individual lesion.

## Results

### Demographic Information

**Figure 1.**
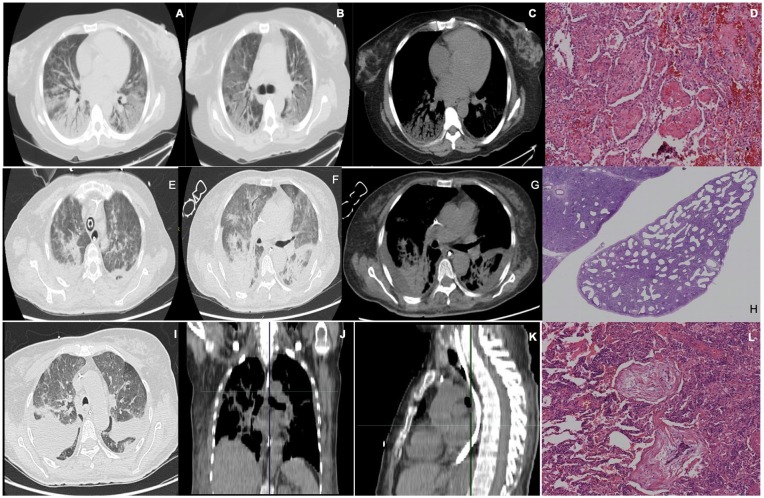
Patient with Classic Hodgkin lymphoma. (A-C) Thoracic computed tomography (CT) scan showing multilobar ground-glass opacities in both lung fields, with condensation of the right inferior lobe. (D) Diffuse alveolar damage in exudative phase. Hematoxylin-eosin (H&E) stain showing an alteration of pulmonary parenchyma with dense eosinophilic hyaline membrane formation and type I pneumocyte necrosis, which detach from the alveolar surface. Patient with acute lymphoblastic leukemia. (E-G) Thoracic CT scan showing diffuse infiltrates and multilobar patch consolidations, bilateral pleural effusions with passive atelectasis, and basal septal effusion. (H) Nonspecific interstitial pneumonia. H&E stain showing a homogeneous alteration of the lung parenchyma with fibrosis, remodeling and smooth muscle proliferation, associated to chronic inflammation, predominantly a fibrosing pattern. Patient with classic Hodgkin lymphoma. (I-K) Thoracic CT scan with extensive parenchymal involvement with areas of consolidation in the right pulmonary field, ground-glass opacities, nodular pleural thickening, pleural effusion, and mediastinal adenomegalies. (L) Organized pneumonia. H&E stain showing infiltration by fibroblasts rich in proteoglycans and myofibroblasts in alveolar lumen, affecting distal bronchioles, alveolar conducts, and peribronchial alveoli.

**Figure 2.**
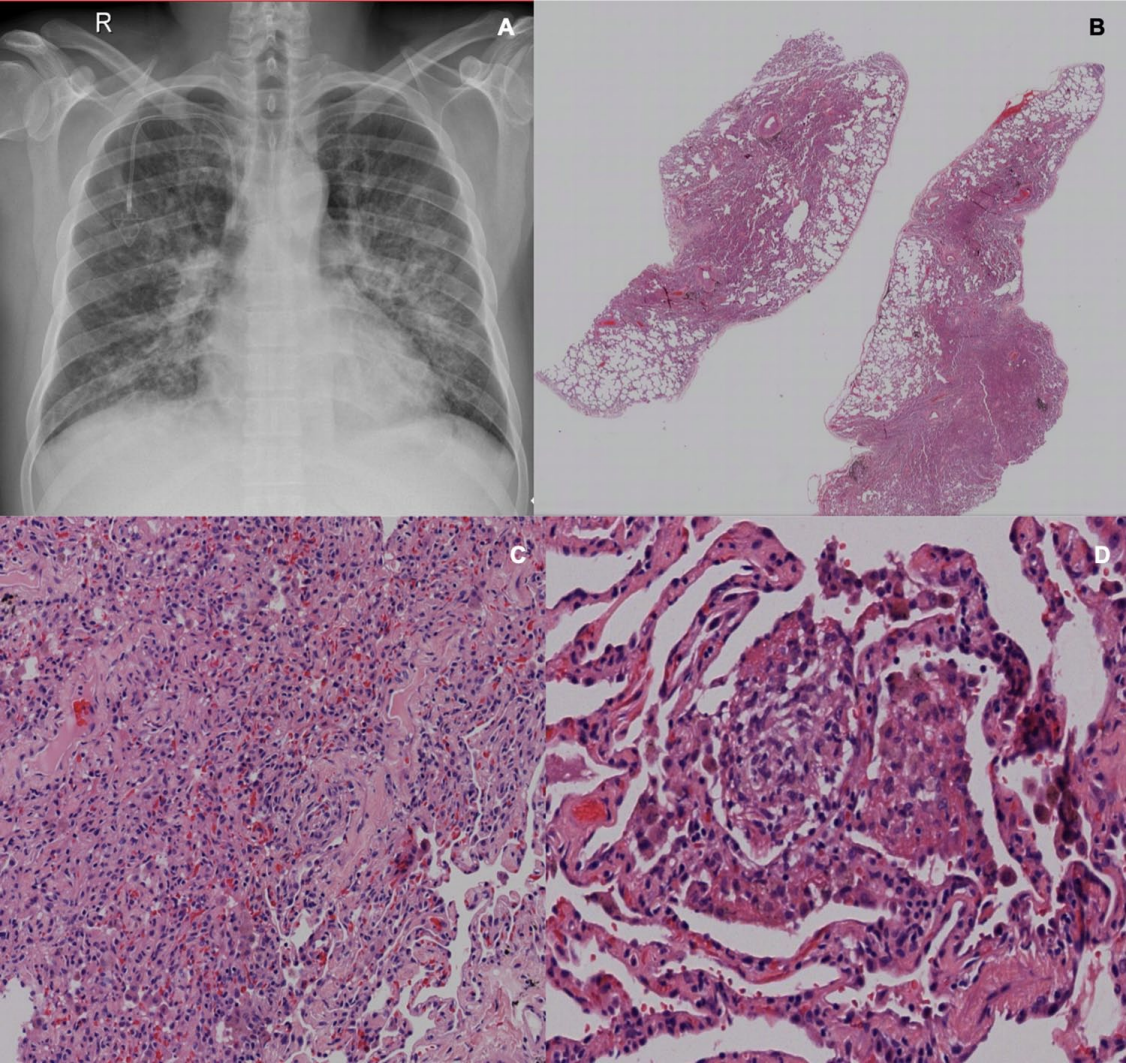
Patient with classic Hodgkin lymphoma. (A) Chest X-ray with bilateral interstitial infiltrates, no evidence of consolidations or pleural effusion. A superior vena cava catheter is observed (R, right side). (B-D) Bleomycin toxicity. Hematoxylin-eosin stain depicting the presence of malformed intra alveolar granulomas, without necrosis or microorganisms. Cultures were negative.

**Figure 3.**
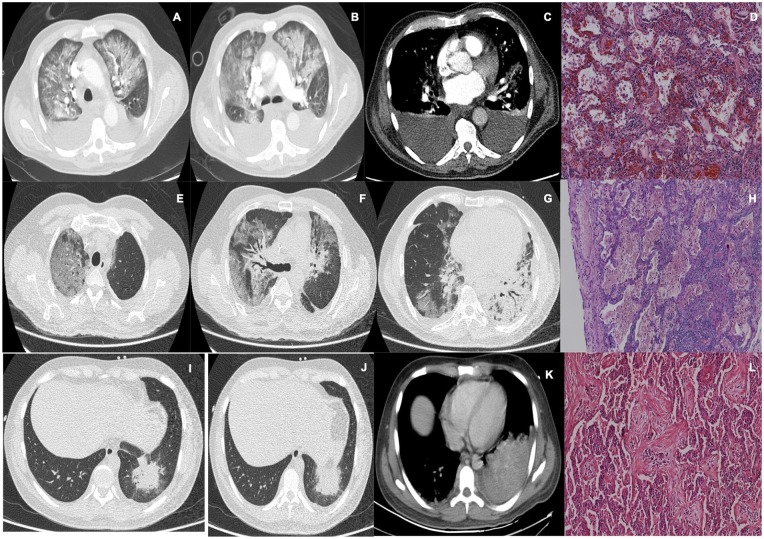
Patient with chronic myelocytic leukemia. (A-C) Thoracic computed tomography (CT) scan with diffuse interstitial infiltrates, septal thickening, ground-glass opacities, and bilateral pleural effusions. (D) Diffuse alveolar damage with alveolar hemorrhage. Hematoxylin-eosin (H&E) stain with alteration in lung parenchyma and presence of hemorrhage on alveolar lumen, associated to dense eosinophilic hyaline membrane formation. Patient with bulky follicular lymphoma. (E-G) Thoracic CT scan with substantial parenchymal compromise, ground-glass opacities in the periphery of superior right lobe, and extensive areas of consolidations. (H) H&E stain showing diffuse alveolar damage in exudative phase. Patient with acute lymphoblastic leukemia. (I-K) Thoracic CT scan with a rounded lesion in the basal segments of the left inferior lobe, with peripheric air bronchogram, which progresses to a complete consolidation of this lobe. (L) H&E stain showing organized pneumonia.

**Figure 4.**
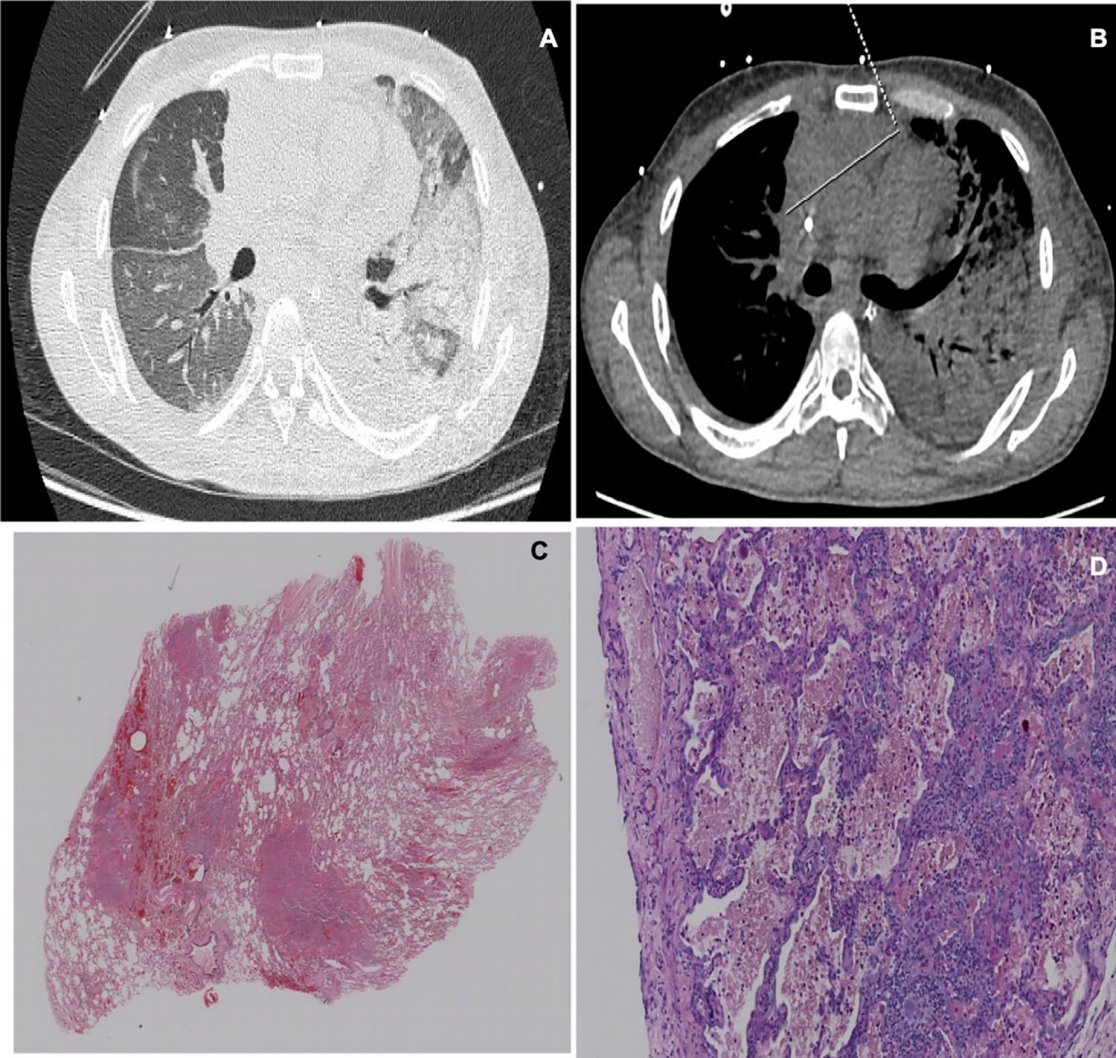
Patient with large B-cell lymphoma. (A, B) Thoracic computed tomography scan showing infiltrates with alveolar occupation, air bronchogram, and complete consolidation of the left superior lobe. (C) Anterior mediastinal mass with measures 11 × 8 × 3 cm. (D) Hematoxylin-eosin stain showing diffuse alveolar damage.

Patients were admitted to the ICU, most frequently for presenting with acute hypoxemic respiratory failure associated with febrile syndrome, febrile neutropenia, or pneumonia (see Table 1 for patients’ overview). Mean age was 47.7 years (SD ±18.16), and APACHE II score at admission was a mean score of 18.6 (SD ±6.7). A thoracic CT scan was performed due to respiratory deterioration showing consolidations in 5 patients, pleural effusion in 5 patients, and ground-glass opacification in 4 patients (Figures 1-4). The most common suspected diagnosis was pneumonia in 6 patients, although alveolar hemorrhage was suspected in 2 patients, progression of oncologic disease with lung compromise in 1 patient, bleomycin toxicity in 1 patient, and septic emboli to the lung from a soft tissue abscess in 1 patient. All but 2 patients were under mechanical ventilation (MV) before the lung biopsy, with a mean time from initiation of MV to lung biopsy of 5.25 days (SD ±4.7). Of the 2 patients without MV prior to lung biopsy, only 1 patient stayed under MV after the procedure for 2 additional days. All of these patients had a complex regimen of antibiotics with or without antivirals and antifungals (Table 2) despite negative cultures, normal BAL, and unremarkable fibrobronchoscopy. Additionally, 5 patients received treatment with corticosteroids. All patients were under MV.

**Table 1.** Patients’ Overview.

	Patient 1	Patient 2	Patient 3	Patient 4	Patient 5	Patient 6	Patient 7	Patient 8
Age/sex	47/female	67/male	17/male	41/male	71/male	55/male	30/male	33/male
Oncologic diagnosis	Classic Hodgkin lymphoma	Acute lymphoblastic leukemia	Classic Hodgkin lymphoma	Classic Hodgkin lymphoma	Chronic myelocytic leukemia	Bulki follicular lymphoma	Acute lymphoblastic leukemia	Large B-cell lymphoma
ICU admission diagnosis	• Acute hypoxemic respiratory failure	• Sepsis of unknown origin • Febrile neutropenia	• Acute hypoxemic respiratory failure • Severe hemorrhagic cystitis	• Acute hypoxemic respiratory failure • Suspected bleomycin toxicity	• ARDS • Pneumonia in immunosuppressed patients • Alveolar hemorrhage	• Pneumonia in immunosuppressed patients	• Severe thrombocytopenia • Febrile syndrome	• Febrile syndrome • Pleural and pericardial effusion
APACHE II score	9	16	21	19	32	20	13	19
Organ dysfunction	Yes	Yes	No	Yes	Yes	Yes	Yes	Yes
Thoracic CT findings	• Multilobe consolidation Diffuse ground-glass opacifications	• Multilobar alveolar infiltrates • Pleural effusion • Posterior atelectasis	• Interstitial pulmonary edema• Bilateral pleural effusion	• Diffuse infiltrates in the 4 quadrants	• Diffuse ground-glass opacification • Bilateral pleural effusion • Posterior atelectasis	• Ground-glass opacification in RUL • Bilateral infiltrates Consolidation in bases	• LLL consolidation with ipsilateral mild pleural effusion	• Ground-glass opacification in LUL with area of consolidation• Bilateral pleural effusion
Pre-biopsy working diagnosis	Multilobar pneumonia	Septic emboli from soft tissue abscess in right upper extremity with pulmonary superinfection	Multilobar pneumonia, mycotic opportunistic infection, alveolar hemorrhage, and PTLD	Opportunistic infection	Multilobar pneumonia, alveolar hemorrhage	Multilobar pneumonia, diffuse alveolar damage	Fungal pneumonia	Pneumonia versus progression of oncologic disease with lung compromise
Time from ICU admission to LB (days)	6	18	30	0	6	11	5	4
LB anatomical site	Right inferior lobe	Right inferior lobe	Right inferior lobe	Right inferior lobe	Right superior lobe	Right superior lobe	Left inferior lobe	Left lingula
Surgical time (minutes)	35	55	30	60	32	18	50	35
Lung biopsy complications								
Pneumothorax	No	No	Yes	No	No	Yes	Yes	No
Persistent pneumothorax	No	No	No	No	No	No	No	No
Hemorrhage that required reintervention	No	No	No	No	No	No	No	No
Mortality related to the procedure	No	No	No	No	No	No	No	No
Histopathological diagnosis	Bleomycin toxicity	Unspecified interstitial pneumonia	Organized pneumonia, alveolar hemorrhage	Bleomycin toxicity	Alveolar hemorrhage	Diffuse alveolar damage	Organized pneumonia	Diffuse alveolar damage
Change in patient’s diagnosis	Yes	Yes	Yes	Yes	No	No	No	Yes
Change in patient’s treatment	Yes	Yes	Yes	Yes	Yes	No	No	Yes
Mortality	Yes	No	No	No	Yes	Yes	Yes	Yes
Cause of death	Multiorgan failure	N/A	N/A	N/A	Multiorgan failure	ARDS fibrotic phase, sepsis	Septic shock	Septic shock
Time from LB to death (days)	9				33	24	46	34

Abbreviations: ICU, intensive care unit; ARDS, acute respiratory distress syndrome; CT, computed tomography; RUL, right upper lobe; LLL, left lower lobe; PTLD, post-transplant lymphoproliferative disorder; LB, lung biopsy.

**Table 2.** Medical Management Prior to Lung Biopsy.

	Patient 1	Patient 2	Patient 3	Patient 4	Patient 5	Patient 6	Patient 7	Patient 8
Glucocorticoids	Yes Methylprednisolone (pulses)	Yes Hydrocortisone	Yes Hydrocortisone	No N/A	Yes Methylprednisolone	Yes Methylprednisolone	No N/A	Yes Methylprednisolone
Antibiotics	Yes Vancomycin Cefepime Moxifloxacin	Yes Doripenem Amikacin Polymixyn B	Yes Meropenem Trimethropin Sulfamethoxazole Polymixyn B	Yes Meropenem Vancomycin	Yes Meropenem Moxifloxacin	Yes Cefepime Vancomycin Moxifloxacin Trimethropin Sulfamethoxazole	Yes Meropenem Vancomycin Amikacin	Yes Meropenem Vancomycin
Antiviral	No	No	No	No	No	No	No	Acyclovir
Antifungal	No	Fluconazole	No	No	No	Fluconazole	Fluconazole	Caspofungin

Abbreviation: N/A, not available.

### Surgical Procedure

The median time from ICU admission to VATS lung biopsy was 6 days (range = 0-30 days). All patients had criteria for ARDS on the day of the lung biopsy, being severe (partial pressure of oxygen/fraction of inspired oxygen [PaO_2_/FiO_2_] ≤ 100 mm Hg) in 2 cases, moderate (100 <PaO_2_/FiO_2_ ≤ 200 mmHg) in 5 patients, and mild (200 < PaO_2_/FiO_2_ ≤ 300 mm Hg) in 1 case. Additionally, they had a positive end-expiratory pressure of at least 5 cm H_2_O, plus diffuse bilateral pulmonary infiltrates not explained by heart failure or volume overload.

Of the 8 patients, 4 required vasoactive support posterior to the lung biopsy, of which 2 had been already on vasoactive support before the procedure. Surgery had a median time of 35 minutes (range = 18-60 minutes). Three patients received red blood cell transfusion before the lung biopsy due to pancytopenia, while none required a transfusion after the procedure. Regarding platelets, 3 patients were transfused before and 1 patient after the biopsy. On the control chest X-ray, 3 patients had small pneumothorax but none had persistent pneumothorax (>48 hours), hemothorax, or hemorrhage that required reintervention. There was no mortality related to the procedure.

### Lung Biopsy Results

Histopathological diagnosis ranged from bleomycin toxicity in 2 patients, alveolar hemorrhage in 2 patients, organizing pneumonia in 2 patients, DAD in 2 patients, and unspecified interstitial pneumonia in 1 patient. All microbiological cultures were negative (Figures 1-4)

### Outcomes

After histopathological diagnosis, 6 patients suffered a modification in their treatment.

Most patients had various antimicrobials schemes throughout ICU stay, so the ongoing regime at the time of lung biopsy results was chosen for analysis. Treatment with steroids was changed in 1 patient and initiated in another. Antimicrobials were ceased in 2 patients and narrowed in 2 patients. Prophylactic acyclovir and fluconazole were started in 1 patient. In 2 of the 8 patients, there were no changes executed in management (Tables 2 and 3). Finally, 5 patients died with a median time from lung biopsy to death of 33 days (range = 9-46 days; Table 1).

**Table 3.** Change in Management After Lung Biopsy.

	Patient 1	Patient 2	Patient 3	Patient 4	Patient 5	Patient 8
Addition y/o change of glucocorticoid	No	Change to methylprednisolone	No	Addition of prednisone	No	No
Antimicrobials changed/ceased	No	No	No	Meropenem Vancomycin changed	Meropenem Moxifloxacin ceased	Meropenem Moxifloxacin ceased
Bacterial cover narrowed	No	No	Meropenem ceased	Initiation of cefepime	No	No
Antiviral initiated/ceased	N/A	N/A	N/A	Initiation of prophylactic acyclovir	No	Acyclovir ceased
Antifungal initiated/ceased	N/A	No	N/A	Initiation of prophylactic fluconazole	No	Caspofungin ceased

Abbreviation: N/A, not available.

## Discussion

Historically, it has been described that patients with hematologic malignancies have poor outcomes when admitted to the ICU. However, recent observational studies have shown improvements in outcomes and availability of treatments, secondary to advances in diagnostic and therapeutic tools.^5^

Among the most common causes for ICU admission are respiratory problems, and it is also the most common cause of complication during ICU stay,^5,6^ leading to ARDS in 10% of patients admitted to the ICU and 23% of mechanically ventilated patients as shown in the study by Bellani et al.^7^ In this study, mortality reached 46% in patients with severe ARDS.^7^ The risk for developing pulmonary infections depends on the degree of immunologic compromise and on patient’s comorbidities and nosocomial exposure,^1^ which explains why they are at the highest risk. It is important to highlight the nondepreciable contribution of noninfectious complications that could also lead to ARDS, such as inflammatory disorders, hemorrhage, treatment-related toxicities, recurrent malignancy, and so on.^8^ All of these entities require an etiological diagnosis for an optimal therapeutic choice, which remains a challenge in this population. The inability to obtain a specific diagnosis highly increases the exposure to unnecessary drugs, favoring adverse effects, bacterial resistance, and negatively affecting patient’s outcomes.^1^

The diagnostic workup to study diffuse pulmonary infiltrates in the context of ARDS include imaging (chest X ray or thoracic CT); microbiologic and immunologic investigations of blood, urine, sputum, and endotracheal aspirate; and invasive techniques such as bronchoscopy to perform BAL. The diagnostic challenge relies on the fact that radiographic abnormalities can overlap between different entities, there is limited usefulness and specificity of biomarkers due to abnormal response to inflammatory processes, and diagnostic yield of cultures and bronchoscopy vary widely.^1^ Concerning bronchoscopy, recent studies showed a great variability in the diagnostic yield for pulmonary infections (between 31% and 74%) in critically ill immunosuppressed patients, probably as a result of delayed sampling time and previous antibiotic use.^6^

This set of patients, similar to the ones presented in this case series, probably benefit from a lung biopsy to obtain a specific diagnosis. Among surgical lung biopsies, VATS procedures have the advantage of being minimally invasive; reducing bleeding, postoperative complications, and pain-related morbidity; and shortening hospital stay. However, it has been reported in some series that in around 25% of cases, the procedure has to be converted to a thoracotomy to obtain adequate sample tissue, for several reasons including technical problems, instrument issues, surgeon inexperience, and so on.^9^ It has been stated by multiple authors that in capable hands VATS lung biopsy has the same diagnostic yield as open thoracotomy.^10^ Nevertheless, VATS lung biopsy has not been widely studied in this context and further randomized controlled trials are required.

Although DAD is considered the histopathological hallmark in patients with ARDS, several studies have shown that only a fraction of patients have DAD. In a retrospective cohort study conducted by Park et al, 36.9% of patients had DAD, while 63.1% presented with non-DAD, mainly diffuse interstitial lung disease (22.7%) and infection (20.2%).^11^ Another study found interstitial lung disease to be the most common histopathological finding in 30% of cases, followed by infection in 20%, while DAD was seen in 31.6%.^12^ Thus, patients can be subclassified by their histopathological findings in DAD-ARDS and non-DAD-ARDS, which has important implications, as there is no specific therapy for DAD but there could be therapeutic options for non-DAD-ARDS.^11^ In our patients, only 2 presented DAD and none had infection. The most common finding was interstitial lung disease (Table 1).

Diagnostic yield, efficacy, and security of surgical lung biopsy, mostly frequently via thoracotomy than VATS, have been analyzed in several observational studies. In our case series, all of the biopsies gave a specific diagnosis and change in management was seen in 6 of the 8 patients. In one study, the diagnostic yield of open lung biopsy in the context of patients with hematologic malignancy was reported at 62%.^8^ Its effectiveness, defined as the ability to generate a change in therapy, has been very variable among studies with reports that go from 57% to 94%^3,8,12,13^; the efficacy obtained in the study performed by Papazian et al indicated that none of the patients received steroids prior to the biopsy.^13^ A recent meta-analysis that included 95 case series of open lung biopsy reported a change in therapy of 78%, being the most common alterations in the initiation or tailoring of therapy, while discontinuation was seen in 10%.^4^ In our patients, the alteration in antimicrobial scheme was the most frequent.

The rate of complications reported in the literature is around 30%, in which the most common complication is persistent pneumothorax and initiation or an increase in ventilatory support.^3,8,12^ One study reported hemorrhagic shock that required reintervention in 2 out of 76 patients.^12^ Furthermore, the meta-analysis by Wong and Walkey reported only one case of perioperative mortality from 512 patients analyzed.^4^ This is consistent with our patients, in which only mild pneumothoraces were seen in 3 patients, with no persistent pneumothoraces or hemorrhagic complications, despite the fact that several patients had severe anemia and thrombocytopenia that required transfusions prior to the biopsy. In fact, only 1 patient required a platelet transfusion posterior to the biopsy. However, studies analyzing open lung biopsy include both VATS and thoracotomy in contrast with our patients, in whom only VATS was performed.

The most consistent finding throughout the literature is regarding 30-day mortality. To our knowledge, most studies have reported a mortality rate around 50%,^3,4,12,13^ and there was no significant difference in mortality between patients who changed their treatment and those who did not.^11^ Only in one study, an improvement in mortality was seen in patients who had a specific biopsy diagnosis (infection, malignancy, and inflammatory disease) compared with those with nonspecific findings (interstitial fibrosis, DAD; 5% vs 38%).^8^ In our series, mortality rate was higher (5 out of 8 patients) but it was not attributable to VATS lung biopsy since there was no perioperative mortality, but to the advanced and critical stage of their diseases. Furthermore, the procedure did not appear to affect mortality in this set of patients.

A recent meta-analysis comparing BAL and lung biopsy in patients with cancer reported a similar diagnostic yield among the 2 procedures, with noninfectious diagnosis being more common in lung biopsy and infectious diagnosis in BAL. Although change in management occurred more frequently in patients with lung biopsy, complications and mortality were significantly higher in this subset of patients.^14^ This is why timing to perform lung biopsy is crucial, and although the optimal time is unknown, there must be a balance between the desire to increase diagnostic yield and performing an invasive procedure that is not without harm.^15^

## Conclusions

Video-assisted thoracoscopic surgery lung biopsy is a minimally invasive procedure that can be done in patients with hematologic malignancy who develop ARDS without a clear etiology. It appears to be a relatively safe procedure with a high histopathological yield, resulting in a modification of treatment in a nondepreciable percentage of patients. Nevertheless, this is a surgical procedure that comes with risks and it should not be done without first exhausting noninvasive/less invasive techniques. Furthermore, although there are minimal mortalities directly related to the procedure, this subset of patients has a high mortality rate, secondary to their underlying disease and critical state, and it is not affected by lung biopsy. Importantly, evidence of this topic comes from observational studies and case series that have major limitations. Further randomized controlled trials are required to obtain solid evidence about the safety and efficacy of thoracoscopic lung biopsy in this subset of patients.
